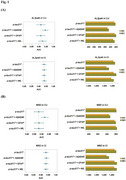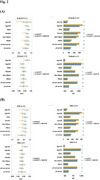# Plasma phosphorylated tau 217 and amyloid‐β 42/40 for amyloid risk in subgroups

**DOI:** 10.1002/alz70861_108025

**Published:** 2025-12-23

**Authors:** Heekyoung Kang, Heejin Yoo, Daeun Shin, Sang Won Seo, K‐ROAD

**Affiliations:** ^1^ Samsung Medical Center, Gangnam‐gu, Seoul Korea, Republic of (South); ^2^ Samsung Medical Center, seoul, seoul Korea, Republic of (South)

## Abstract

**Background:**

Alzheimer’s disease (AD) is characterized by the accumulation of amyloid‐β (Aβ) pathology. Recently, plasma biomarkers, particularly *p* ‐tau217, have emerged as promising tools for early diagnosis and risk stratification. In this study, we evaluated the diagnostic performance of *p* ‐tau217 combined with other plasma biomarkers in distinguishing Aβ Positron emission tomography (PET) positivity in cognitive unimpaired (CU) and cognitively impaired (CI) individuals across diverse clinical subgroups.

**Method:**

We analyzed 2,497 participants from the Korea‐Registries to Overcome dementia and Accelerate Dementia (K‐ROAD) cohort, including 636 CU and 1,971 CI individuals. Plasma *p* ‐tau217 was measured using both SIngle MOlecule Array (SIMOA) and Meso Scale Discovery (MSD) assays, alongside Aβ42/40, Glial fibrillary acidic protein (GFAP), and Neurofilament light chain (NfL). Aβ PET positivity was determined using the MRI‐based regional direct comparison Centiloid (rdcCL) method. We assessed the diagnostic performance of biomarker combinations through the area under the receiver operating characteristic curve (AUC), Akaike Information Criterion (AIC) and Bayesian Information Criterion (BIC), and performed subgroup analyses based on age, sex, body mass index (BMI), and Apolipoprotein E (APOE) ε4 status.

**Result:**

In CU individuals, the combination of *p* ‐tau217 and Aβ42/40 significantly improved diagnostic accuracy (AUC: ALZpath 0.944 vs. 0.914, MSD 0.920 vs. 0.879, *p* < 0.001) and model fit (AIC /BIC, *p* < 0.0001) compared to *p* ‐tau217 alone. In contrast, in CI individuals, the combination provided only modest improvements in model fit without significantly enhancing AUC. GFAP and NfL did not contribute significantly to amyloid detection in either group. Subgroup analyses in CU individuals showed the greatest improvements in older adults, females, and APOE4 non‐carriers, regardless of obesity status. In CI individuals, the combination had no significant impact on AUC except in males, where a small but significant increase was observed (p = 0.002).

**Conclusion:**

Combining *p* ‐tau217 with Aβ42/40 enhances amyloid detection in CU individuals, improving both diagnostic accuracy and model fit, whereas its impact in CI individuals is limited. These results highlight the potential of plasma biomarker combinations for refining early AD diagnostics and individualized risk assessment.